# The Cross-Talk Between the Peripheral and Brain Cholesterol Metabolisms

**DOI:** 10.3390/cimb47020115

**Published:** 2025-02-11

**Authors:** Ilinca Savulescu-Fiedler, Luiza-Roxana Dorobantu-Lungu, Serban Dragosloveanu, Serban Nicolae Benea, Christiana Diana Maria Dragosloveanu, Ana Caruntu, Andreea-Elena Scheau, Constantin Caruntu, Cristian Scheau

**Affiliations:** 1 Department of Internal Medicine, The “Carol Davila” University of Medicine and Pharmacy, 050474 Bucharest, Romania; 2 Department of Internal Medicine and Cardiology, Coltea Clinical Hospital, 030167 Bucharest, Romania; 3 Department of Cardiology, Emergency Institute for Cardiovascular Diseases “C.C. Iliescu”, 022328 Bucharest, Romania; 4 Department of Orthopaedics, “Foisor” Clinical Hospital of Orthopaedics, Traumatology and Osteoarticular TB, 021382 Bucharest, Romania; 5 Department of Orthopaedics and Traumatology, The “Carol Davila” University of Medicine and Pharmacy, 050474 Bucharest, Romania; 6 Department of Infectious Diseases, The “Carol Davila” University of Medicine and Pharmacy, 050474 Bucharest, Romania; 7 Departament of Infectious Diseases, National Institute for Infectious Diseases “Prof. Dr. Matei Balș”, 021105 Bucharest, Romania; 8 Department of Ophthalmology, Faculty of Dentistry, The “Carol Davila” University of Medicine and Pharmacy, 050474 Bucharest, Romania; 9 Department of Ophthalmology, Clinical Hospital for Ophthalmological Emergencies, 010464 Bucharest, Romania; 10 Department of Oral and Maxillofacial Surgery, “Carol Davila” Central Military Emergency Hospital, 010825 Bucharest, Romania; 11 Department of Oral and Maxillofacial Surgery, Faculty of Dental Medicine, “Titu Maiorescu” University, 031593 Bucharest, Romania; 12 Department of Radiology and Medical Imaging, Fundeni Clinical Institute, 022328 Bucharest, Romania; 13 Department of Physiology, The “Carol Davila” University of Medicine and Pharmacy, 050474 Bucharest, Romania; 14 Department of Dermatology, “Prof. N.C. Paulescu” National Institute of Diabetes, Nutrition and Metabolic Diseases, 011233 Bucharest, Romania; 15 Department of Radiology and Medical Imaging, “Foisor” Clinical Hospital of Orthopaedics, Traumatology and Osteoarticular TB, 021382 Bucharest, Romania

**Keywords:** cholesterol, 24-hydroxycholesterol, 27-hydroxycholesterol, statins, PCSK9i, metabolism, physiology, physiopathology, brain, peripheral metabolism, preoperative management

## Abstract

Cholesterol is an essential element for the development and normal function of the central nervous system. While peripheral cholesterol is influenced by liver metabolism and diet, brain cholesterol metabolism takes place in an isolated system due to the impermeability of the blood–brain barrier (BBB). However, cross-talk occurs between the brain and periphery, specifically through metabolites such as oxysterols that play key roles in regulating cholesterol balance. Several neurodegenerative conditions such as Alzheimer’s disease or Parkinson’s disease are considered to be affected by the loss of this balance. Also, the treatment of hypercholesterolemia needs to consider these discrete interferences between brain and peripheral cholesterol and the possible implications of each therapeutic approach. This is particularly important because of 27-hydroxycholesterol and 24-hydroxycholesterol, which can cross the BBB and are involved in cholesterol metabolism. This paper examines the metabolic pathways of cholesterol metabolism in the brain and periphery and focuses on the complex cross-talk between these metabolisms. Also, we emphasize the regulatory role of the BBB and the need for an integrated approach to cholesterol management.

## 1. Introduction

All plasma membranes of eukaryotic cells contain sterols. Cholesterol synthesis is considered an evolutionary step from prokaryotes to eukaryotes, as long as cholesterol synthesis does not occur in the absence of oxygen [1]. Each cell of the human body is capable of synthesizing cholesterol [2], with endogenous cholesterol representing approximately 90% of the body’s total cholesterol; this grounds the assertion that cholesterol is a fundamental compound of the human body and essentially involved in cell functions and physiological processes [3]. Cholesterol is found mostly but not exclusively in intracellular organelles, as well as in plasma membranes, contributing to cell membrane fluidity, permeability, and integrity. Cholesterol is also found in the blood in plasma lipoproteins, which represent the major blood cholesterol carriers.

Cholesterol is presented in unesterified and esterified versions, with different distributions throughout the body. Most of the cellular cholesterol is unesterified and found mainly in plasma membrane leaflets, representing 20–25% of lipid molecules within these structures [4,5,6]. However, cholesterol is also found, in small amounts, in the endoplasmic reticulum (ER), Golgi apparatus, and nucleus. Some plasma membrane cells contain specialized microdomains rich in cholesterol, known as lipid rafts or lipid domains [7,8]. The circulating forms of cholesterol are represented by esterified cholesterol, found mainly in low-density lipoproteins (LDLs) (about 60%) but also in very low-density lipoproteins (VLDLs) and high-density lipoproteins (HDLs). LDLs transport cholesterol from the liver to the cells, while HDLs transport cholesterol in the opposite direction, from the tissues to the liver [9].

Oxidized cholesterol derivatives (i.e., oxysterols) are excretion cholesterol products also involved in cholesterol homeostasis and represent metabolic intermediates in steroid hormones, vitamin D3, and bile acids biosynthesis [10].

The widely used paradigm is that higher cholesterol levels are strongly associated with higher cardiovascular morbidity and mortality. However, the optimal range for total cholesterol levels associated with the lowest incidence of cardiovascular and overall events remains a topic of debate and continuously evolves based on clinical evidence.

The first point to emphasize regarding “optimal” cholesterol levels is that these levels fluctuate with age. Early life, especially while suckling, represents a critical phase in brain growth and myelination [11,12]. Plasma cholesterol levels are physiologically elevated at this time, rising quickly across all species, highlighting its crucial role in these processes [13]. Deficits in cholesterol synthesis during this period may lead to significant abnormalities in brain development, with major functional consequences [14]. On the contrary, in adults, hypercholesterolemia is associated with increased cardiovascular risk and general mortality [15].

In 2019, the European Society of Cardiology and the European Society for Atherosclerosis Research reported that the target for LDLs varies according to the cardiovascular risk, as follows: very high risk *<* 55 mg/dL, high risk *<* 70 mg/dL, moderate risk *<* 100 mg/dL, and low risk < 116 mg/dL [16]. To reach these targets, aggressive and sustained lipid-lowering treatments are mandatory [17].

In adulthood, both hypercholesterolemia and low cholesterol levels are linked to increased general and cardiovascular mortality, as well as to detrimental behaviors, including aggression and suicidal tendencies [18,19,20,21,22].

A long-term follow-up study showed that sustained low serum cholesterol for more than two decades is related to the most increased level of mortality [23], including high rates of cancer-associated deaths. Treatment with statins was also shown to increase cancer incidence in several studies [24,25,26].

A large United States cohort study involving 30,687 patients followed for 15 years (between 1999 and 2014) [27] found that the relationship between plasma cholesterol levels and all-cause mortality is not linear; instead, it follows a U-shaped pattern. This indicates that both high and low cholesterol levels are associated with increased general and cardiovascular mortality. The study concluded that low cholesterol levels correlate with higher mortality rates, with total cholesterol levels below 200 mg/dL strongly linked to general mortality, and levels below 120 mg/dL significantly associated with higher mortality and cardiovascular morbidity. Conversely, cholesterol levels of 280 mg/dL or higher were primarily related to deaths from cardiovascular diseases. The same study [23] established cutoff values for total cholesterol of 172 mg/dL, 267 mg/dL, and 205 mg/dL for all-cause mortality, respectively cardiovascular disease mortality and cancer mortality. Another interesting conclusion was that people with higher education levels were more likely to have higher total cholesterol values. While it is often perceived that lower education correlates with poorer health outcomes, this finding might indicate that those with higher education may be more prone to certain dietary habits or lifestyle choices that increase cholesterol levels. Also, individuals with a higher level of education often have better access to healthcare information and resources, but this does not necessarily mean healthier choices are made [23].

## 2. Cholesterol Level Regulation

Sterols are essential for membrane structure, fluidity, and permeability; therefore, their concentration within plasma membranes needs to be tightly regulated [28]. This constant level is tissue-specific and maintained through a dynamic equilibrium between the accumulation and excretion of cholesterol [29].

### 2.1. Cholesterol Biosynthesis and Uptake

**Figure 1:**
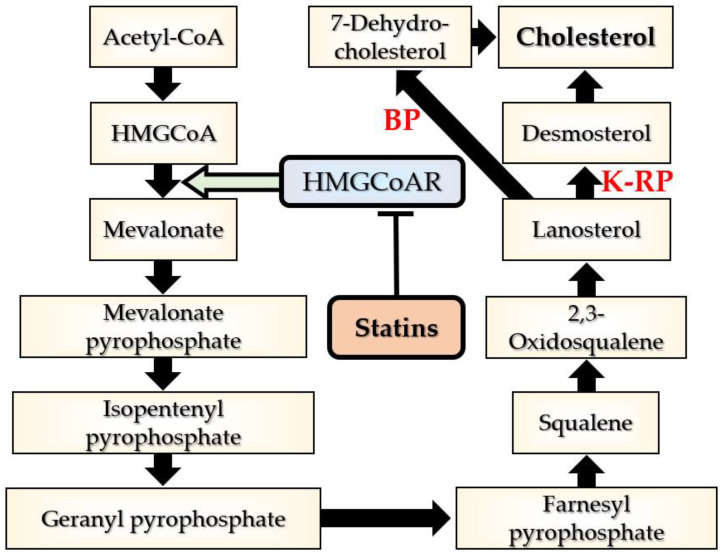
The mevalonate pathway leading to cholesterol synthesis. Green arrow = catalytic role; Acetyl-CoA = acetyl coenzyme A; BP = Bloch pathway; HMGCoA = β-hydroxy β-methylglutaryl-CoA; HMGCoAR = HMG-CoA reductase; K-RP = Kandutsch–Russell pathway.

Cholesterol originates from the following two sources: endogenous and exogenous. The endogenous source is dominant and is represented by cell biosynthesis from acetyl coenzyme A under the action of 3-hydroxy-3-methylglutaryl coenzyme A reductase (HMGCoAR). The essential element in cholesterol synthesis is represented by mevalonate [30]. Mevalonate is converted in a few steps to lanosterol, after which cholesterologenesis occurs differently for neurons and astrocytes; in neurons, cholesterol derives from 7-dehydrocholesterol (on the Kandutsch–Russell pathway) while in astrocytes from desmosterol (on the Bloch pathway) [31] (Figure 1) [30]. The other source that ensures up to 22% of total plasma cholesterol is represented by food [32]. Cholesterol biosynthesis occurs via the isoprenoid pathway which also produces other active molecules, such as coenzyme Q10 (CoQ10), heme-A, and dolichol, with important cellular functions. Briefly, CoQ10 and heme-A are involved in aerobic cellular respiration and in adenosine triphosphate generation, CoQ10 being particularly important as an antioxidant, and dolichol is required for glycoproteins synthesis [33].

Cholesterol synthesis is regulated by HMGCoAR, a rate-limiting enzyme in the mevalonate–cholesterol biosynthetic pathway which presents a sterol-sensing domain sensitive to sterol levels within the ER membrane. The cross-talk between HMGCoAR and ER is essential for cholesterol synthesis. With increased sterol levels, HMGCoAR interacts with the ER-resident protein insulin-induced gene (INSIG), undergoing proteasomal degradation [34,35,36].

Sterol regulatory element-binding proteins (SREBPs) transcription factors are located on the ER membrane and modulate the synthesis and uptake of cholesterol depending on the levels of intracellular sterol content [37,38].

Circulating cholesteryl esters enter the cell via LDL receptors (LDLrs) and scavenger receptor class B type 1 (SR-B1). LDLrs are responsible for binding VLDLs and LDLs, which contain apolipoproteins such as apoE and apoB100. SR-B1 is found in hepatocytes and endocrine cells and binds HDL particles that contain ApoA1 apolipoprotein [39,40,41,42]. ApoA1 is largely distributed in the cerebrospinal fluid (CSF), despite not being produced or located in the brain [43]. ApoA1 is delivered to the CSF at the level of the choroid plexus and is involved in cholesterol transfer from the peripheral nervous system [44,45]. Low levels of ApoA1 and HDL were correlated with lower cognitive performances [46].

Hydrolysis of cholesteryl esters contained in LDLs generates free cholesterol delivered to cellular compartments via membrane proteins involved in cholesterol trafficking in mammals, called Niemann–Pick disease types C1 and C2 (NPC1 and NPC2) [47,48].

### 2.2. Cholesterol Excess Regulation: Cholesterol Efflux and Cholesterol Stores

Because of the cellular toxicity of cholesterol accumulation, the excessive cholesterol needs to be excreted (unesterified or as oxysterols) or stored as cholesteryl-ester droplets.

There are several mechanisms involved in cellular cholesterol efflux. The central nervous system tissue expresses various types of ATP-binding cassette (ABC) transporters, such as ABCA1, ABCG1, and ABCG4, all expressed by neurons [49]. ABCA1 are expressed in higher levels in neurons, as well as in glial cells [50,51,52]. ABC transporters excrete unesterified cholesterol from the membrane or cellular cytosol to the external environment [29].

Acting as cholesterol receptors, the extracellular apolipoproteins [32] uptake the cholesterol under a chemical gradient [53,54]. ApoA1-containing lipoproteins are taken up by various receptors belonging to the LDLr family, highly expressed in glial cells (astrocytes), endothelial brain capillary cells, and neurons [29,55,56,57,58].

Oxysterols are products of cholesterol oxidation [59], more polar than cholesterol and, subsequently, more prone to being absorbed by cells compared to cholesterol [60]. They also activate LXRs, leading to a reduction in the intracellular cholesterol content [61]. Oxysterols seem to be stronger promoters of atherosclerosis than cholesterol itself [62,63,64]. Atherosclerotic lesions develop more rapidly in animals fed with a diet high in oxidized fatty acids and oxidized cholesterol [63,65]. Oxysterols are generated through enzymatic cholesterol oxidation (e.g., 24S-hydroxycholesterol (24-OHC) in the brain, 27-hydroxycholesterol (27-OHC) in several tissues, and 7-hydroxycholesterol (7-OHC) in the liver) or are generated from non-enzymatic cholesterol oxidation upon oxidative stress (e.g., 25-hydroxycholesterol (25-OHC), cholesterol 5β6β-epoxide, 7-ketocholesterol, and 7β-hydroxycholesterol) [66,67,68]. Oxysterols are transported mainly by HDLs, unlike cholesterol which is transported mainly by the LDL/VLDL system [69]. Oxysterols play a crucial role in cholesterol transport out of cells and are essential for maintaining cholesterol homeostasis. Acting as ligands for SREBP2, oxysterols downregulate endogenous cholesterol synthesis, through a mechanism similar to statins, decreasing HMGCoAR expression [70]. They also have various other roles, including the contribution to bile acid synthesis following uptake by the liver.

The most studied metabolites of cholesterol are 24-OHC, 25-OHC, and 27-OHC. 27-OHC is the most abundant plasmatic cholesterol metabolite and is generated in peripheral circulation from cholesterol enzymatic oxidation under the action of the CYP27A1 enzyme. 24-OHC is found peripherally, reflects only the brain cholesterol turnover, and is generated exclusively in the central nervous system (CNS) [71,72]. Plasmatic levels of 24-OHC are increased in high brain cholesterol turnover and low in neurodegenerative processes [73]. 25-OHC is produced in all cells, in response to cholesterol-enriched foods.

24-OHC, 25-OHC, and very small amounts of 27-OHC that cross the blood–brain barrier (BBB) are present in the brain. Brain 27-OHC levels are very low if the BBB is intact [73].

The interaction between 24-OHC and 27-OHC in the brain plays a particularly important role in maintaining brain cholesterol homeostasis [74,75,76,77,78]. At the moment, this relation is a topic of debate; some authors support the hypothesis that 27-OHC downregulates brain cholesterol synthesis and 24-OHC production [79], while other studies enforced the opposite concept, that 27-OHC, via stimulating CYP46A1 activity, increases the 24-OHC levels [80].

The ratio between 24-OHC and 27-OHC (24-OHC/27-OHC) in serum or plasma is considered a marker for cholesterol metabolism [81]. The ratio of 27-OHC to 24-OHC is functionally significant and is tightly regulated. 24-OHC/27-OHC have differing metrics in different brain areas [82].

## 3. Oxysterols Within the Brain

### 3.1. 27-Hydroxycholesterol

27-OHC is generated in both hepatic and extrahepatic tissues. Within the brain, cholesterol is converted to 27-OHC to a lesser extent than to 24-OHC, because CYP27A1 is expressed at lower levels in neurons, astrocytes, and oligodendrocytes [83]. In the brain, CYP7B1 converts 27-OHC into 7alpha-hydroxy-3-oxo-4-cholestenoic acid (7-HOCA) [84].

If the BBB is intact, the level of 27-OHC in the CSF reflects the plasmatic 27-OHC levels [85]. Low plasmatic cholesterol levels are followed by decreased 27-OHC production and reduced 27-OHC influx into the brain [86]. On the contrary, hypercholesterolemia is associated with increased levels of 27-OHC, including within the CNS [87]. Higher levels of 27-OHC within the brain are related not only to the increased BBB permeability [88] but also to higher CYP27A1 expression in glial cells [89].

Peripheral injection of 27-OHC in rats upregulates ABCA1 in the brain and downregulates HMGCoAR and LDLr [75].

27-OHC has numerous deleterious effects on CNS through various mechanisms. Firstly, increasing levels of 27-OHC downregulate HMGCoAR, decreasing brain cholesterol levels. At the CNS level, 27-OHC reduces brain cholesterol synthesis and also 24-OHC production [90]. Secondly, 27-OHC facilitates the transport of cholesterol between astrocytes and neurons leading to its accumulation in neurons [91]. Moreover, 27-OHC promotes neuroinflammation [92], which holds special relevance regarding Alzheimer’s disease (AD). The detrimental effect of neuroinflammation is sustained by the observation of a direct relationship between neuronal and astrocytic inflammatory markers, such as IL-1, IL-6, and tumor necrosis factors alpha and amyloid beta (Ab) levels [93]. Another notable effect of 27-OHC is that it induces overactivation of the brain renin–angiotensin-aldosterone system (RAAS) [94] and increases the oxidative stress [95], with negative consequences on cognition. Brains of deceased individuals with AD displayed elevated levels of 27-OHC compared to the brains of those who died without the diagnosis of AD [82].

Most 27-OHC results from processed and ultra-processed animal food (mainly microwave and grill preparation), excessive baking, cooking, or heating of cooked refrigerated products [96,97]. 27-OHC is inversely correlated with HDL levels [98].

### 3.2. 24S-Hydroxycholesterol

As 24-OHC results exclusively from brain cholesterol oxidation under 24-hydroxylase (CYP46A1) action, its plasmatic and CSF levels are directly correlated to brain cholesterol metabolism [84]. However, the level of 24-OHC (cerebrosterol) is influenced not only by the brain cholesterol level but also by the number and integrity of neurons. The CSF levels of 24-OHC more accurately reflect its expression because of the independence of hepatic clearance [85]. In healthy individuals, 24-OHC plasma levels are maintained throughout most of their lifetime. After the seventh decade of life, as a consequence of a natural decline in neuronal mass, serum levels of 24-OHC decrease [84]. In the early stages of neurodegenerative diseases, as a consequence of myelin loss, 24-OHC levels are elevated but decrease in advanced stages [99]. 24-OHC traverses the BBB almost integrally with only a very small percent (1%) entering the CSF [72,100].

24-OHC has important contributions in cholesterol elimination and synthesis, and in cholesterol transport between astrocytes and neurons [101]. Another important function of 24-OHC is modulating NMDA receptors, contributing to synaptic plasticity and learning [102,103,104], of particular importance in hippocampal neurons [105].

**Figure 2:**
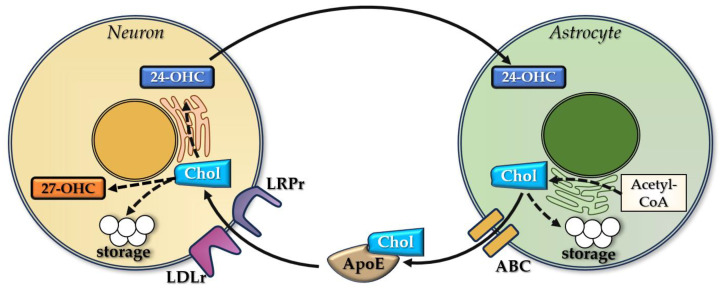
The role of 24-OHC in cholesterol transport between astrocytes and neurons.

Neuronal cholesterol levels are primarily influenced by the efflux of cholesterol from astrocytes because of significant limitations in cholesterol production in neural cells [106]. 24-OHC migrates from neurons to astrocytes, activating LXR and its target genes ABCA1 and ABCG1, which cause the efflux of ApoE-bound cholesterol from astrocytes. ApoE is released by astrocytes under stimulation from 24-OHC and will bind cholesterol, which is subsequently taken up by neurons [107]. 24-OHC binds to α and β LXR receptors [108,109], inhibiting brain cholesterol synthesis mainly in astrocytes [110]. LXR receptor activation determines astrocyte synthesis of ApoE and ABCA1/ABCG1, stimulating the transport of cholesterol from the neurons to the astrocytes [107,111] (Figure 2). LXR activation increases ApoE expression in neurons and also induces ABCA1/ABCG1 expression in the endothelial cells of brain capillaries, as shown by experimental studies [112].

In normal human brain tissue, 24-hydroxylase is primarily expressed in neurons, which are the main site for cholesterol turnover [75]. It is also present in lower concentrations in oligodendrocytes and microglia [113]. Moreover, the highest CYP46A1 levels are found in cortical, hippocampal, and cerebellar neurons [80,114,115], suggesting either special metabolic rates or a particular sensitivity to high cholesterol levels of the neurons located in these regions. Decreased 24-hydroxylase activity (experimentally through administration in mice of voriconazole, an inhibitor of CYP46A1) is followed by cognition improvement, likely due to preservation of hippocampal cholesterol [116,117]. But also, 24-hydroxylase hyper-expression is linked to neuronal cholesterol loss and impaired cognition, as seen in aging or stress [118]. In mice deficient in 24-hydroxylase, cholesterol excretion significantly slows, and its synthesis is suppressed. Furthermore, noticeable deficiencies in motor learning related to cognitive function may be identified [119]. Oxidative stress induces CYP46A1 hyperexpression [120].

There is only an apparent contradiction between the 24-hydroxylase levels, 24-OHC levels, and cognition, as 24-OHC functions as a double-edged sword; low levels are harmful to cognition, while excessive accumulation can become neurotoxic [121]. These observations support the concept that brain cholesterol homeostasis is finely tuned, especially in cerebral regions engaged in higher cognitive functions. Therefore, brain cholesterol metabolism is in a continuous adjustment toward equilibrium.

24-OHC plays a dual role for neurons, acting as a pro-survival or pro-death factor, depending on its concentration. At physiological concentrations, 24-OHC is neuroprotective, through LXRs activation [68,122]. This effect is lost at higher concentrations, due to the inhibition of LXR transcriptional activity [122], becoming a promoter of neuronal loss [123,124]. In older people, cholesterol synthesis and 24-OHC levels are concordant and decreased at the hippocampal level [125], explaining the low performances in learning and memory [119,126].

24-OHC has beneficial effects, such as anti-inflammatory and anti-atherogenic properties [127], but also deleterious effects, inducing cell death at high concentrations [73]. In cultured cells, 24-OHC promotes the non-amyloidogenic processing of soluble amyloid precursor protein (APP) [128,129] and impairs neuronal accumulation of hyperphosphorylated tau protein [130]. Toxic accumulation of oxysterols within the brain is fostered in conditions that increase BBB permeability, the most common conditions being aging and hypercholesterolemia, both linked to oxidative stress [131].

### 3.3. 25-Hydroxycholesterol

25-OHC is produced mainly in microglia [132] and regulated by the activity of cholesterol 25-hydroxylase (CH-25H) [133], an enzyme localized in the ER. Higher cholesterol levels in the ER lead to higher 25-OHC levels, in return inhibiting several intracellular cholesterol mechanisms, such as degradation of HMGCoAR, prevention of SREBP2, and activation of LXR receptors [29,34,134]. These actions explain why 25-OHC exhibits neuroprotective effects, limiting neuroinflammation and neuronal loss, improving synaptic transmission and lipid rafts formation [135].

**Figure 3:**
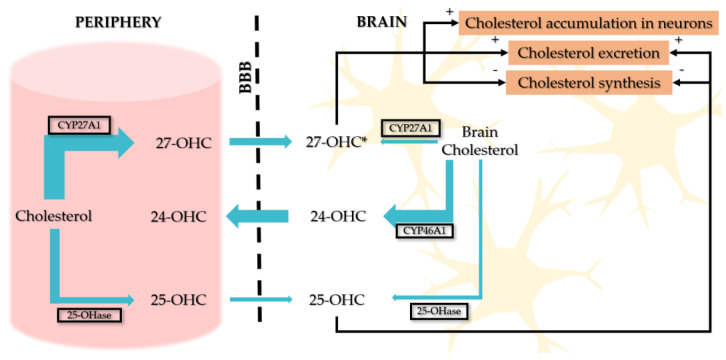
The exchange of cholesterol between plasma and brain via the BBB. Cholesterol homeostasis in neurons. * 27-OHC levels depend on the BBB’s integrity; hypercholesterolemia increases the BBB’s permeability and CYP27A1 in glial cells.

25-OHC has the capacity to cross membranes and vascular barriers [85] (Figure 3).

Through the action of sulfotransferases, cholesterol and oxysterols become cholesterol derivatives with increased water solubility, like cholesterol sulphate and oxysterol 3-sulfates, respectively [136]. These sulphated derivatives exhibit neuroprotective effects, such as stimulation of neuroprotective neurosteroids synthesis [137], and limit brain oxysterol accumulation [138]. Levels of oxysterol sulphate are significantly reduced in the prefrontal cortex of patients with AD [138].

## 4. Brain Cholesterol Metabolism

### 4.1. Role of Cholesterol in the Central Nervous System

In humans, but also in other species (e.g., primates and mice), total body cholesterol is ~2200 mg/kg body weight (2.2 mg/g fresh tissue) [28]. In all species, the brain’s cholesterol concentration is approximately 15–20 mg/g of brain matter [14,139,140,141,142,143,144], but the amount of brain cholesterol differs among species and is significantly greater in humans. The organ with the highest cholesterol content is the adult human brain, where it accounts for approximately 25% of the body’s total cholesterol [84,111].

Cholesterol is the major lipid compound in the brain [139,145], and it is not equally distributed, differing among brain regions [146].

In hypercholesterolemia, the permeability of the BBB is increased, resulting in higher plasma levels of 24-OHC compared to individuals with normal plasma cholesterol levels. However, in hypercholesterolemic patients, higher 27-OHC levels and the cross-talk between 24-OHC and 27-OHC contribute, to some extent, to increased 24-OHC plasma levels [147]. Therefore, a reasonable conclusion is that the separation between the CNS and the body is largely restricted to cholesterol molecules, not cholesterol metabolism.

There are significant differences in the metabolic and turnover rates of cholesterol between the periphery and the brain. In the periphery, the basal metabolic rate of cholesterol and the cholesterol turnover rate are directly related to each other. In contrast, while the metabolic rate of cholesterol in the CNS is high, its turnover rate is slow, at around 0.03% per day compared, with 0.7% per day in the periphery [145]. Another notable difference is between the short half-time of plasma cholesterol (of few days) [14,145], and the half-time of brain cholesterol, which ranges between 6 months and 5 years [111,148,149]. This is yet another indication that for proper functioning, the brain requires a stable cholesterol level.

The observed differences between the adult and the very young animal brains refer not only to turnover rates, low in the adult brain and high during the development period but also to the type of cholesterol. In the adult brain, 99.5% of cholesterol is unesterified [150], unlike the very young animal brain, where the amount of cholesteryl esters is higher [140,141,142,143,144].

While a large part of the literature suggests that cholesterol metabolism in the adult brain is very slow, some studies indicate that it might not be as static as previously thought [151,152]. Nonetheless, it is still slower than in younger individuals, where both cholesterol synthesis and degradation are more active [153].

### 4.2. Regulatory Mechanisms of Cholesterol Metabolism Homeostasis

Most of the adult brain’s cholesterol (around 70–80%) is found in myelin sheaths, while the remaining quantity is identified in astrocytes and plasma membranes of neural cells [14]. The concentration of cholesterol is low in the cytosol and CSF [150].

The majority of brain cholesterol originates from local synthesis in the ER within the nerve cells [154,155], with highly increased levels during the early period of development, when myelination and neuronal growth are essential [144]. Following these events, the production of cholesterol carries on at significantly lower rates [156]. Unlike early life, when neurons are the primary source of brain cholesterol, after myelination, glial cells become the main source of cholesterol in the brain [144]. Cholesterol homeostasis relies on the cross-talk between astrocytes (main producer) and neurons (main consumer) in the adult brain [31]. The astrocyte-synthetized cholesterol is carried out by membrane ABC transporters. After, cholesterol is taken up by ApoE and ApoI lipoproteins (also synthetized by astrocytes), which further interact with their receptors belonging to LDLr and LRP, localized on neuronal plasma membranes. The final step of this cross-talk is neuronal internalization of cholesterol [75,157]. ApoE is the main apolipoprotein transporter in the CNS, being a key player in brain cholesterol metabolism. The main source of ApoE is represented by astrocytes [158].

Both astrocytes and neurons use glucose and lactate as a metabolic source for cellular processes, but neurons prefer the oxidative metabolism [159] over lactate production which is primarily used by astrocytes as an energy source [160]. These two pathways are complementary and essential for glutamate–glutamine metabolism [161].

Alongside regulating the BBB, astrocytes play complex roles in numerous brain functions, as well as neural growth and support [162,163]. The neurovascular unit is composed of astrocytes alongside endothelial cells, pericytes, and vascular smooth muscle cells; this unit is essential for the regulation of cerebral blood flow and underlines the critical function played by the astrocytes [164].

### 4.3. Role of the Blood–Brain Barrier in Brain Cholesterol Metabolism

In the periphery, cholesterol needs are met through the following two main sources: de novo synthesis and circulating lipoproteins. Conversely, the pool of cholesterol in the CNS is mainly supplied by local synthesis and recirculation.

CNS cholesterol metabolism is tightly regulated [165] to maintain a stable absolute brain cholesterol level throughout life [33]. BBB integrity plays a key role in the preservation of brain cholesterol and the metabolic autonomy of the brain. BBB prevents cholesterol uptake from the systemic circulation; therefore, brain cholesterol metabolism relies on recycling [166].

Cholesterol is essential for neuronal functions, and astrocytes play a major function in brain cholesterol homeostasis. Neuronal cholesterol is sufficient for survival and growth but insufficient for building efficient synapses, essential for all physiological brain functions, including learning, memory, and behavior [150,167]. For this last purpose, neurons need extra-neuronal cholesterol provided by astrocytes [168,169,170].

Cholesterol is a key component of myelin [165] and a capital element for dendrite formation and synapse development [171]. It is also important for mitochondrial function and neurotransmitter receptor expression [172], exhibiting neuroprotective effects [173] and antioxidant properties [171].

Maintaining normal levels of brain cholesterol is required for proper brain function, making the regulation of excess cholesterol crucial to this need. Cholesterol excess is prevented through active secretion (excretion) and storage mechanisms.

Cholesterol is removed daily from the brain across the BBB either as hydroxycholesterol derivatives (daily elimination of 6–7 mg of 24-OHC) [72] or as ApoA1- and ApoE-bound cholesterol in the CSF (1–2 mg/day) [174,175]. Cholesterol efflux from neurons to ApoA1 and ApoE [49] is mediated by ABC transporters, resulting in lipoproteins that are released into the CSF [176,177,178] and selectively taken up by LDL receptor-related protein 1 (LRP1), mainly expressed in neurons [29] and SR-B1 receptors expressed in endothelial cells within brain capillaries [55,56]. ApoA1 is found in HDL, and HDL particles can cross the BBB [179,180] while ApoE cannot.

ApoE represents the main mechanism of cholesterol transport between astrocytes and neurons [181], as well as a pathway for cholesterol excretion into the CSF [182]. The highest ApoE expression level is recorded in the liver, followed by the brain [183]. Under normal conditions, neurons only produce small amounts of ApoE, while the main source of ApoE (around 80%) is represented by astrocyte cells [184]. During stress or injuries, neuronal ApoE production may increase [185].

Within the CNS, astrocytes express LDLrs, which are an important functional receptor for ApoE and Ab [186,187,188]. ApoE is involved in Ab degradation and clearance [189] and plays a role in mitochondrial dysfunction [181,189]. Of the various isoforms, ApoE4 is linked to a higher risk for the development of AD and shows detrimental effects on BBB permeability [190,191].

A minor (less than 1%) part of the cholesterol surplus is stored in lipid droplets as esterified cholesterol [192]. Cholesterol esterification occurs as a result of cholesterol acyltransferase activity; while these enzymes are expressed in glial cells, they are mainly present in neurons and are sensitive to higher free cholesterol levels [193].

The free exchange of cholesterol between plasma and the brain is prevented by an intact BBB. It was shown that cholesterol derivatives can cross the BBB [194], while lipoproteins and free cholesterol do not exhibit this ability [72,111]. However, only an intact BBB restricts the passage of free cholesterol.

The BBB impermeability to lipoproteins was demonstrated more than 50 years ago. Connor [195] characterized the peripheral and brain cholesterol metabolism by prelabeling the sterol in the yolk sac and administrating C^14^ cholesterol to hens. In newborn chickens, the labeled sterol was found largely in peripheral tissues (95–98% from the reference yolk sac level) but also in the brain tissue in small amounts (around 11% of the reference level). We note that there are significant differences between the two percentages but also that the brain-specific activity was not null, underlining the importance of cholesterol biosynthesis in the brain. Another study, on terminally ill patients, showed that administration of C^14^-labeled cholesterol is followed by the detection of approximately 3% of the labeled cholesterol in CNS structures [196]. One alternative explanation for the flow across the BBB might be represented by cholesterol transport at the choroid plexus [197].

The permeability of circumventricular organs (CVOs) is dependent on the molecule size, as shown by murine immunohistochemistry studies. The permeability differs according to the low molecular weight of the tracer, and molecules heavier than 10 KDa cannot cross the barrier [198,199,200]. While the molecular weight of cholesterol is only 386.7 g/mol or 0.3867 kDa, there remains the question of whether free cholesterol can pass through CVOs and reach the brain tissue [93,201,202]. There are limited data regarding this hypothesis as long as the permeability of statins across CVOs is also incompletely understood.

Brain endothelial cells express large amounts of LDLr, SR-B1, and ABCA1 [55,203], which raises the possibility that small amounts of CNS cholesterol originate in the plasma. The primary source of cholesterol in the brain is its synthesis within the CNS, although a small amount of brain cholesterol does originate from plasma [204]. Suckling animals have high levels of circulating lipoproteins during the accumulation of sterol in the brain [14]. This may hint at the possibility that lipoproteins can cross the BBB; however, there are limited data in this regard.

The paradigm is that brain and peripheral cholesterol homeostases are independent. Some authors modulate this paradigm, concluding that brain and peripheral cholesterol metabolism are largely independent but not completely isolated, sustaining the existence of low cholesterol transport from the circulation into the brain [205,206].

Experimental murine studies have demonstrated that cholesterol can cross the BBB in small amounts, a process that is amplified in hypercholesterolemia [207]. Rabbits receiving a high-fat diet for 10 weeks presented higher BBB permeability compared to rabbits fed a normal diet [208]. Therefore, the BBB’s permeability enables peripheral cholesterol to pass through [209].

The increase in BBB permeability in hypercholesterolemia translates to higher plasma 24-OHC levels compared to persons with normal plasma cholesterol levels. However, in hypercholesterolemic patients, higher 27-OHC levels and the cross-talk between 24-OHC and 27-OHC may partly contribute to increased 24-OHC plasma levels [210].

The most reasonable conclusion is that the separation between the CNS and the periphery is largely restricted to the cholesterol molecule, not to its metabolism. Brain cholesterol metabolism is tightly regulated but integrated into general body metabolic processing.

## 5. The Effects of Lipid-Lowering Therapy on Brain Cholesterol Metabolism

### 5.1. Statins and Central Cholesterol Regulation

Statins decrease cholesterol synthesis by inhibiting HMGCoAR, upregulating LDLr, and increasing LDL-cholesterol (LDL-C) clearance via apolipoprotein B-rich hepatic absorption facilitation [211]. Statins also reduce triglyceride-rich lipoprotein biosynthesis in the liver [212]. Within the brain, the highest levels of HMGCoAR are recorded in cholinergic cortical and hippocampal neurons [213].

Downregulating the mevalonate pathway is accompanied by a decrease in molecules produced, including CoQ10, and non-steroid isprenoid compounds (e.g., farnesyl pyrophosphate (FPP) and geranylgeranyl pyrophosphate (GGPP)) [154].

CoQ10 plays a major role in cellular integrity and ATP formation and has antioxidant properties [214,215,216]. The highest level of CoQ10 is found in tissues with high metabolic activity, such as the heart, brain, muscles, and kidneys [217,218]. The circulating form of CoQ10 mainly binds to LDLs and VLDLs, while small amounts are bound to HDLs [219]. Although it is unclear whether CoQ10 can cross the BBB, most studies conclude that CoQ10 can flow to the brain side only if the tight junctions of the barrier are disrupted. Meanwhile, in an integer BBB, there is no transport of CoQ10 across the barrier [220].

While dolichol inhibition is related to various myopathies, neuropathies, and diabetes [214,221], it is not obvious to what extent the adverse effects of statins are related to brain cholesterol reduction or to the reduction in other mevalonate metabolites [222]. Statins exhibit numerous intricate effects such as anti-inflammatory and antioxidant properties [118,223,224,225,226]. However, statins also present deleterious effects on the CNS, such as impairing synaptic vesicle release and reducing synapse density after exposure to low concentrations of lovastatin for a short period of 7–14 days [227]; blocking dendritic growth and dendritic retraction [228]; promoting apoptosis [229]; reducing astrocyte viability after only 3 days of administration [230]; reducing ApoE expression [185]; and increasing serotonin uptake, through direct effects on serotonin transporters [231].

The effects of statins in the CNS primarily depend on their structure and molecular weight, as well as the presence of specific transporters [232,233,234]. Also relevant for their ability to cross the BBB is their concentration, with higher levels associated with increased permeability [235].

Statins may be either lipophilic (atorvastatin, simvastatin, cerivastatin, pitavastatin, fluvastatin, and lovastatin) or hydrophilic (rosuvastatin and pravastatin), and their ability to affect the synthesis of brain cholesterol depends on their capacity to cross the BBB. While lipophilic statins easily pass through the BBB, hydrophilic statins only manage to traverse it in small quantities. Not all lipophilic statins have the same BBB permeability, with simvastatin and fluvastatin having higher BBB penetration capacities (around 25%) followed by atorvastatin [233,236].

Atorvastatin and simvastatin are defined as potent lipophilic statins and cross the BBB, suppressing brain cholesterol synthesis with subsequent decreases in plasma 24-OHC levels [237]. Decreases in circulating levels of 24-OHC were also observed for less lipophilic statins (lovastatin) and some hydrophilic statins (pravastatin), thus implicating another mechanism that is probably related to circulatory LDL clearance [127].

The central effects of statins depend on the treatment’s duration. Most studies concluded that short-term statin treatment has no effect on the levels of cholesterol in the brain [238], but long-term treatment does. Statin administration for more than 6 months was shown to decrease cholesterol levels in the CSF [239]. One study evaluating the effects of simvastatin on cholesterol and 24-OHC levels in patients with hypercholesterolemia evaluated the effects at 6 and 24 weeks [237]. The results show that hepatic cholesterol synthesis (evoked by the plasma lathosterol/cholesterol ratio) was reduced similarly at the two timepoints, and so were the plasma 24-OHC levels and 24-OHC/total cholesterol ratio [73].

### 5.2. Statins and Potential Effects on Cognition

It is considered that statins promote cardiovascular and cerebrovascular health through their exhibited antioxidant and anti-inflammatory actions [240]. However, it is unclear whether statins yield beneficial or adverse effects on cognition. While most authors consider that initiating statin therapy in late life does not negatively impact cognition, it is uncertain whether long-term administration has favorable effects.

Statins increase the levels of LRP1 at the BBB [241] while also increasing the binding capacity for LDLs, ApoE, and Ab [242]. Conversely, statins decrease FPP and GGPP levels [243], with opposite effects on cognition. Lower FPP levels are associated with beneficial effects on cognition, acting as a protective mechanism against glutamate toxicity [244,245]. However, GGPP reduction leads to serotonin uptake [231], demonstrating how statin administration may lead to depression and anxiety, and, on a structural level, to neurite loss [119,126,246].

Another effect linked to lower isoprenoid levels are the promotion of endothelial protection [247,248], alongside the anti-inflammatory [244], antithrombotic, and antioxidant effects [249]. Statins also exhibit antiapoptotic effects. Simvastatin upregulates the antiapoptotic gene Bcl2, which is essential for neuronal survival [250].

There is a lack of consensus regarding the relationship between cognitive function and plasma lipids [251,252,253,254]. Observational studies and randomized controlled trials in AD have shown contradictory results regarding statin administration [255,256,257]. This might be explained by the heterogeneity of studies but also by the high dependence on individual factors. It might be possible that dementia and, possibly, AD are not associated with statin intake as reported in a study on 18,846 subjects over 65 years old [208].

Cholesterol is involved in the metabolism of APP, with the processing occurring at the level of lipid rafts [258,259]. High-fat diet administration in animal models of AD was shown to promote the synthesis of Ab [207,260,261,262,263,264,265,266] and impede the production of APP [267]. Conversely, low cholesterol levels were followed by the inhibition of β-secretase activity (involved in Ab production) and an increase in α-secretase activity, a proteolytic enzyme involved in APP metabolism [268].

A connection between cognitive impairment and increased levels of LDL and triglycerides was identified [269,270]. The risk of AD occurrence in patients with dyslipidemia varies according to age; higher cholesterol levels during midlife are associated with late-life AD [271]. A high-cholesterol diet contributes to increased amyloid concentration in the hippocampus, associated with oxidative stress and memory deficits [272]. This demonstrates how lowering cholesterol levels through statins in midlife might decrease the risk of AD later in life [273]. However, studies on older adults showed a positive association between LDL levels and cognitive performance [274]. A recent longitudinal study involving patients with AD underlined that LDL-C in higher concentrations translates to improved cognitive function [275]. Conversely, lower cholesterol levels in late life correlate with cognitive deterioration [268,276,277]. Higher cholesterol levels in older individuals are associated with lower risks for AD [278,279].

Importantly, multiple studies have indicated that lower cholesterol levels are associated with violent behavior, including aggression, suicide, and homicide [18,20,280,281]. In the Cardiovascular Prevention Program study, which included 1365 participants, dyslipidemia seems to not be associated with cognitive impairment [282].

The effects of statins on brain cholesterol levels are under debate. Murine studies assessed variable concentrations of simvastatin or pravastatin, and high concentrations were followed by a reduction in plasma cholesterol and lathosterol (an indicator of endogenous cholesterol synthesis), as well as a reduction in fraction synthesis rate (FSR) of cholesterol synthesis in the brain [145]. However, the brain can only maintain cholesterol homeostasis either through suppression of cholesterol outflow, by increasing HMGCoAR activity, or through concordant decreases in 24-OHC and lathosterol [283].

Numerous studies, including the MIRAGE study, have found that statins are linked to a lower risk of dementia and a slower progression of AD [284,285,286,287]. High doses of rosuvastatin or atorvastatin over long periods of time reduce the incidence of dementia [288], as shown by a longitudinal study with a follow-up period of 12 years. Statins may have positive effects on cognitive functions, including verbal memory, logical reasoning, and working memory [273,289]. The positive effects on cognition, particularly in women, may primarily be attributed to the beneficial effects of statins on the vascular system [290].

Other clinical trials [24,291,292] found no decrease in the risk of dementia in patients treated with statins. The Heart Protection Study concluded that dementia occurs with comparable incidence in patients receiving either simvastatin or placebo. The PROSPER (Pravastatin in Elderly Individuals at Risk of Vascular Disease) trial also revealed that cognitive function is no different in patients using statins compared with ones not undergoing lipid-lowering treatment; there was also no proof that statins could prevent vascular dementia or alleviate AD progression [293]. Additionally, statin administration, regardless of the lipophilic quality, had no influence on dementia, as shown by the Three-City with a 7-year follow-up [294].

The greatest benefit of statin in delaying AD progression is obtained by patients under 65 years old and who started statin therapy in midlife [295], while poorer benefits are recorded in patients older than 65 years [296]. However, a contradictory finding is that high doses of statins in diabetic patients or the use of statins in ApoE4 carriers are associated with an increased risk of dementia [297,298].

Simvastatin administration for 7 days triggered a significant decrease in dopamine levels at the level of the nigrostriatum in murine studies [299]. This leads to the question of whether the chronic use of statins is associated with a decrease in dopamine in specific brain synapses [228].

In 2012, the Food and Drug Administration (FDA) announced that statins cause reversible cognitive impairment [300]. Anyway, the FDA updated the beneficial statin effects on cognition. The JUPITER (Justification for the Use of Statins in Primary Prevention) trial warned of a higher risk for reversible decline in cognitive function [273].

Problems in cognition typically arise within a year after the debut of statin treatment [86]. Fortunately, cognitive impairment is short-term and quickly reversible (3 weeks) after statin treatment discontinuation [301] or statin replacement (exchange of a lipophilic statin with a hydrophilic one [302]). There are reports of a recurrence of cognitive impairment upon reintroducing statins after discontinuation [303]. Cognitive changes are more pronounced in potent and lipophilic statins, such as simvastatin and atorvastatin [273,304], and affect patients with a history of alcohol abuse or psychiatric disease to a greater extent [273]. Cognitive impairment appears to be dosage-dependent [301], with better diffusion into the CNS and more impactful effects on brain cholesterol synthesis at higher doses. Alongside their effect on cholesterol synthesis, statins might be responsible for cognitive impairment through their detrimental effects on brain mitochondrial function [301], via lowering CoQ10 levels. Overall, the reductions in CoQ10 and CNS cholesterol may contribute to the cognitive impairment associated with statin use observed in some patients. However, a clear conclusion on the beneficial or deleterious effects of statins on cognitive performance cannot be drawn at this point. Studies are reporting no evidence of statins affecting cognition [305,306,307].

### 5.3. Novel Agents and Their Effects on Central Cholesterol Metabolism and Cognition

Because lowering LDL-C directly correlates with cardiovascular risk reduction, actual recommendations are to decrease LDL-C to new target levels. This is possible because of newer agents, such as ezetimibe or proprotein convertase subtilin/kexin type 9 (PCSK9) inhibitors like evolocumab, alirocumab, or inclisiran [308].

PCSK9 catalyzes LDLr breakdown and determines a decrease in the number of LDLrs [309]; this effect occurs mainly in hepatocytes, where the highest levels of PCSK9 are expressed [310,311]. Within the brain, PCSK9 is only expressed in neurons [312], with the highest levels observed during the developmental period [297], and contributes to cholesterol levels’ stabilization within the CSF by reducing the expression of LDLr, LRP1, VLDLr, and ApoE [313,314,315].

PCSK9 and PCSK9 monoclonal antibodies do not traverse the intact BBB [316,317]. In increases in BBB permeability, PCSK9 traverses to the CNS and proceeds to LDLr degradation [318,319], decreasing the capacity for internalization of LDL-containing particles. PCSK9 interacts with multiple proteins involved in neuronal metabolism and functions, such as neuronal differentiation, migration, and survival.

PCSK9 inhibitors (PCSK9i) increase circulating LDLrs and lead to a robust LDL-C reduction of approximately 60% in monotherapy [320,321]. PCSK9i are indicated mainly for the reduction in LDL-C levels in patients who do not tolerate statins or when statins cannot control LDL levels. PCSK9i enhances LDLr capacity and lowers serum concentrations of 24-OHC, 27-OHC, and cholesterol itself, mainly affecting total cholesterol and 27-OHC levels, and, to a lesser degree, 24-OHC. Therefore, PCSKi does not alter the ratio between 27-OHC and cholesterol, but 24-OHC/total cholesterol and 24-OHC/27-OHC are significantly increased [210].

PCSK9 might be involved in the pathogenesis of AD [318]. PCSK9 is higher in the CSF of AD patients [322,323], with the highest levels being detected in ApoE4 carriers [323]. The expression of PCSK9 in the frontal cortex was higher in patients with AD than in controls [324,325], and several mechanisms could be involved. Firstly, PCSK9 interferes with neuronal cholesterol uptake [326]. Other possible mechanisms are represented by the pro-apoptotic effect of PCSK9 [327,328], neuronal cholesterol depletion (by decreasing ApoE and LDLr), reduction of Ab clearance [324], and the pro-inflammatory effect on cerebral cells exerted by PCSK9 [329,330,331,332]. Conversely, the absence of PCSK9 is associated with better hippocampal spatial memory [325]. PCSK9 levels are also higher in patients with depression [323,333], correlating with depression score [334].

Most analyses of PCSK9i on cognitive functions show no effect on cognition using this medication class. The FOURIER trial (Further Cardiovascular Outcomes Research with PCSK9 Inhibitors in Subjects with Elevated Risk) compared evolocumab with placebo for the secondary prevention in patients on optimized statin therapy, receiving or not ezetimibe; the study revealed no adverse cognition events in patients taking evolocumab [320]. A derived sub-study, named EBBINGHAUS (Evaluating PCSK9 Binding Antibody Influence on Cognitive Health in High Cardiovascular Risk Subjects), was the first to assess cognitive changes associated with PCSK9i as an endpoint and showed no adverse cognitive events in young patients over a 19-month follow-up period [154].

OSLER-1 and OSLER-2 studies compared evolocumab added to standard therapy vs. standard therapy in patients with hypercholesterolemia. They reported that neurocognitive adverse events were more frequent in patients taking evolocumab compared to standard therapy alone [335].

The efficacy and safety of alirocumab compared to statins at the maximal tolerated dose were analyzed in the ODYSSEY trial [336]. The ODDYSEY LONG TERM trial included patients with heterozygous familial hypercholesterolemia with or without cardiovascular disease on an optimized statin dose [337]. The study concluded that neurocognitive adverse events were more frequent in patients receiving alirocumab for 70 weeks than those that received a placebo, including symptoms such as confusion, amnesia, and memory impairment [325]. In the ODYSSEY COMBO I study, alirocumab was compared to a placebo, over 52 weeks, in patients receiving an optimized statin dose, and no difference between the study groups was recorded in regard to neurocognitive adverse events [338].

MEMOGAL was a prospective study that included adult patients with a first prescription of evolocumab or alirocumab, followed for 24 months for cognitive impairment; it showed no changes in neurocognitive performances and no differences between the two PCSK9i in this respect [339].

The effects of evolocumab and alirocumab on cerebral oxysterol production were analyzed after 1 month and 3 months of treatment. In the first assessment, 27-OHC was significantly lower while 24-OHC/27-OHC increased, and the ratio reached even higher levels after 3 months of treatment [81,210]. This finding might reflect an increased brain cholesterol synthesis.

The Individual Case Safety Reports (ICSRs) of the pharmaco-vigilance database on the secondary neuropsychiatric effects of evolocumab and alirocumab were analyzed. Close to 23% of cases showed nervous or psychiatric symptoms, the majority of which were severe. Approximately 60% of patients were not taking concomitant medication, while the rest were taking other drugs including statins or ezetimibe [340].

Oxysterols detected in the peripheral blood correlate with cholesterol absorption, as well as cholesterol synthesis [90,341]. One study found that 24-OHC, 27-OHC, and 25-OHC, along with other sterols, have a direct relationship with seric levels of campesterol and lathosterol; they also concluded that 27-OHC is positively regulated by cholesterol synthesis, while 24-OHC and 25-OHC correlate with cholesterol absorption and synthesis [342].

Ezetimibe limits the intestinal absorption of cholesterol by blocking the Niemann–Pick C1-like 1 transporter [343]. This transporter is involved in oxysterol level regulation through intestinal dietary oxysterol absorption [344].

### 5.4. Lipid-Lowering Therapies and Neurodegenerative Diseases

The most prevalent neurodegenerative diseases are AD and vascular dementia. Diets rich in saturated fats were associated with a higher risk of developing AD [345], while a Mediterranean diet decreased this risk [346]. However, the potential connection between total cholesterol or LDL levels and AD is still under debate.

Recent studies demonstrated a direct correlation between cholesterol levels and cognitive performance [347]. Conversely, there are data showing that persistent increased cholesterol levels do not associate with a higher risk of cognitive impairment compared with decreased cholesterol levels [348]. Another hypothesis states that cholesterol variation, regardless of the direction, leads to cognitive decline; therefore, this should be accounted for in the management of patients with major risks for vascular disease [348].

As previously mentioned, oxidized cholesterol metabolites can cross the BBB and abnormal levels of oxysterols in the brain are associated with increased Ab production [349] and increased neuroinflammation [350]. Gut microbiota plays a role in regulating neuroinflammation and lipid metabolism, and recent data emphasize the cholesterol-lowering effects of probiotics [351,352]. Modulation of gut microbiota or the administration of probiotic supplements may be a promising approach in the prevention of cognitive decline and determent or delay of AD [353]. Murine administration of the SLAB51 probiotic is associated with a decrease in HMGCoAR activity, with subsequent upregulation of LXRs expression within the brain and in the liver and significant decreases in plasmatic and central 27-OHC concentrations [354].

It is clear from the above findings that the nuanced interplay between lipid-lowering therapies and brain cholesterol metabolism imposes the need for further research to clearly establish the long-term neurocognitive implications and to refine management strategies for cholesterol-related disorders. While statins have caused controversy regarding their effects on cognition and brain function, to date PCSK9i did not exhibit a significant risk for the same adverse cognitive effects in recent studies. An overview of the lipid-lowering therapies is provided in Table 1 [16,355].

## 6. Future Research Directions

LXRs agonists could be instrumental in the modulation of cholesterol metabolism with effects on AD based on their ability to attenuate amyloid pathology and microglial inflammation, as demonstrated in animal models [356]. However, the beneficial effects are overweighed by fatty acid and triglyceride upregulation [357,358].

Some statins, such as lovastatin, simvastatin, and pravastatin, are derivatives of microbial cultures, and this fact supports the increased interest for the study of microbiota in respect to dyslipidemia and cognitive functions [359]. Experimental data show that fecal microbiota transplantation might be effective against cognitive decline in animals exposed to a high-fat/high-cholesterol diet [360]. Fermented dairy products present numerous benefits alongside the reductions in cholesterol levels, such as antioxidant, anti-tumoral, and anti-inflammatory effects [361]. Short-chain fatty acids may play a role in the pathogenesis of AD and are involved in the maintenance of BBB integrity as well as neuroinflammation inhibition [362].

Future directions in lipid-lowering therapy include plant foods, marine algae, probiotics, and prebiotics to respond to both needs to lower cholesterol level and to improve cognition.

Healthy diets are increasingly promoted and plant foods have shown effectiveness in cardiovascular disease prevention due to their low amounts of fats and bioactive compounds, which act as cholesterol-lowering agents via different mechanisms [363]. Among them, soy proteins impair cholesterol absorption, lowering liver cholesterol levels and reducing the expression of several genes regulating lipid transport proteins [364]. Dietary fibers decrease cholesterol levels because of their capacity to bind and excrete bile acids while decreasing cholesterol absorption; short-chain fatty acids formation contributes to this effect, increasing cholesterol excretion and inhibiting cholesterol production [365]. Polysaccharide-based ingredients such as b-glucans exhibit beneficial properties in terms of cholesterol metabolism and are accepted by the European Food Safety Agency and the FDA [366]. These compounds conduct to bile salts sequestration and to active moieties resulted from polysaccharide fermentation by gut microbiota [367].

Marine algae exhibit neuroprotective effects through their bioactive compounds [368]. An increasingly studied molecule with hypolipidemic properties is naringenin (Nar) [369]. Citrus juices, especially orange, grapefruit, and bergamot juices, are rich in Nar, which inhibits acyl-coenzyme A cholesterol acyltransferase, HMGCoAR, and PCSK9 [370,371,372,373,374]. Beneficial effects on the lipid profile were confirmed for bergamot juice, which lowers total cholesterol, LDL-C, and triglycerides and increases HDL levels, as shown in animal models [375], as well as in patients with dyslipidemia [376,377]. Overall, the use of Nar in lowering cholesterol levels may represent an effective and novel approach that is not burdened by the adverse effects related to statins or PCSK9i.

## 7. Conclusions

Cholesterol plays a central role in CNS development and regulation, with a tightly regulated metabolism. The impermeability of the BBB keeps brain cholesterol operating within an isolated system. However, some metabolites such as oxysterols, enable essential cross-talk between brain and peripheral cholesterol systems, serving as key regulators of cholesterol balance.

Neurodegenerative conditions, including Alzheimer’s and Parkinson’s diseases, imply disruptions of this balance and bring attention to the importance of maintaining proper cholesterol homeostasis. The regulatory role of the BBB is essential in these processes, given its influence in determining both the passage of oxysterols and the broader interplay between brain and peripheral cholesterol. BBB permeability increases in various conditions, most commonly represented by aging and hypercholesterolemia, both associated with toxic accumulation of oxysterols within the brain.

The effects of lipid-lowering therapies raise critical considerations. While statins and PCSK9 inhibitors are effective in managing hypercholesterolemia, their impacts on brain cholesterol and cognitive health require further investigation.

An integrated approach taking into account the interdependence of brain and peripheral cholesterol systems is essential. It is expected that future research will improve our understanding of these complex pathways, advance cholesterol management, and address associated neurological outcomes.

## Figures and Tables

**Table 1:** Overview of lipid-lowering therapy and implications for cholesterol management [16,355].

Lipid-Lowering Therapy	Average LDL-C Reduction	Indication	Mechanism of Action	Adverse Effects
Moderate-intensity statins	30% (monotherapy)	First-line treatment for lipid-lowering therapy and ASCVD risk reduction	↓ Hepatic cholesterol production by blocking HMGCoAR → ↑ LDLr expression → higher uptake of LDLs from the bloodstream	Myopathy Rhabdomyolysis Hepatotoxicity Diabetes mellitus Hemorrhagic stroke Proteinuria
High-intensity statins	50% (monotherapy)	Recommended up to the highest tolerated dose to reach the goals set for the specific level of risk		
Ezetimibe	65% (in combination with high-intensity statins) 85% (in combination with high-intensity statins and PCSK9 inhibitors)	Second-line therapy in association with statins when the therapeutic goal is not achieved at the maximal tolerated statin dose or when statins cannot be prescribed	Blocks intestinal absorption of dietary and biliary cholesterol → ↑ LDLr expression → ↑ LDL clearance from the bloodstream	Gastrointestinal adverse effects
Bile acid sequestrants	18–25% (monotherapy) 10–25% further decrease in LDL-C in combination with statins 18% further decrease in LDL-C when added to statins and ezetimibe	Second-line therapy in association with statins when the therapeutic goal is not achieved at the maximal tolerated statin dose or in cases of statin intolerance in combination with ezetimibe. Third-line therapy as an addition to statin plus ezetimibe therapy when the therapeutic goal is not achieved	Prevent the reabsorbtion of both the drug and cholesterol in the blood by binding the bile acids in the intestinum → liver synthesizes more bile acids from hepatic cholesterol → ↑ demand for cholesterol and a ↑ LDL-R expression	Gastrointestinal adverse effects Increased circulating TG levels (contraindicated in baseline TG > 400 mg/dL) Affects the absorption of many drugs and fat-soluble vitamins
PCSK9 inhibitors	60% (monotherapy) 75% (in combination with high-intensity statin) 85% (in combination with high-intensity statin plus ezetimibe)	Third-line therapy for the following: • Primary prevention in patients at very high risk but without FH if the LDL-C goal is not achieved on a maximum tolerated dose of a statin and ezetimibe • Secondary prevention for patients at very high risk not achieving their goal on a maximum tolerated dose of a statin and ezetimibe • Very-high-risk FH patients who do not achieve their goal on a maximum tolerated dose of a statin and ezetimibe If a statin-based regimen is not tolerated at any dosage, added to ezetimibe	PCSK9 binds to the LDL-R and promotes its degradation → ↑ LDL concentration in the plasma. PCSK9 inhibitors increase LDL-R expression by reduction in the plasma levels of PCSK9 → ↑ clearance of LDLs → decrease in LDL-C levels PCSK9 inhibitors also decrease Lp(a) levels	Itching, erythema, swelling, pain at the site of injection Allergic reactions—flu-like symptoms Increased risk of new, onset diabetes mellitus or neurocognitive dysfunction have been suspected but not demonstrated Occurrence of antidrug antibodies—very rare
Inclisiran	No data	Investigational	Stimulates the catalytic breakdown of PCSK9 mRNA in hepatocytes → reduction in hepatic synthesis of PCSK9 → increase in LDL-R expression → increased clearance of LDLs → decrease in LDL-C levels	Injection-site adverse reactions
Bempedroic acid	21.4% (monotherapy) 18% (in combination with statins) 38% (in combination with ezetimibe)	Reducing the risk of myocardial infarction and coronary revascularization in adults at risk and unable to take statins therapy. Adjunct to diet in combination with other LDL-C lowering therapies or alone when concomitant LDL-C lowering therapy is not possible	Inhibits ACLY which catalyzes the formation of acetyl-CoA → decreased cholesterol synthesis in liver → increased LDL-R expression → decrease in LDL-C levels	Hyperuricemia Tendon rupture Renal toxicity Cholelithiasis Benign prostatic hyperplasia Combination with simvastatin or pravastatin causes an increase in the concentrations of these drugs and, therefore, may increase the risk of myopathy
Mipomersen	No data	Adjunct to lipid-lowering medications and diet for the treatment of statin-intolerant patients with severe HoFH	Inhibits of ApoB 100 production in the liver by binding with ApoB mRNA and preventing its translation → lowering LDL and VLDL levels	Adverse reactions at the injection site Liver toxicity
Lomitapide	30–50% (dose-dependent)	Indicated as once daily oral treatment for lowering LDL-C levels in adults with HoFH	Inhibits MTP → hinders the production of VLDLs in the liver and chylomicrons in the intestine	Gastrointestinal adverse effects Reduced absorption of fat-soluble vitamins and essential fatty acids Hepatotoxicity
Fibrates	50% reduction in the TG levels <20% reduction in the LDL-C levels	Indicated for patients with elevated TG levels and low HDL-C levels	Agonists of PPARs, acting via transcription factors regulating various steps in lipid and lipoprotein metabolisms	Renal dysfunction Liver disease Gallbladder disease Increased risk of pancreatitis

↑—increase/stimulate, ↓—decrease/inhibit, →—leads to; ApoB—apolipoprotein B; ACLY—ATP-citrate lyase; ASCVD—atherosclerotic cardiovascular disease; FH—familial hypercholesterolemia; HDL-C—HDL cholesterol; HoFH—homozygous familial hypercholesterolemia; HMG-CoAR—enzyme 3-hydroxy-3-methylglutaryl coenzyme A reductase; LDL-C—low-density lipoprotein cholesterol; LDL-Rs—receptors; Lp(a)—lipoprotein A; MTP—microsomal triglyceride transfer protein; mRNA—messenger RNA; PCSK9—proprotein convertase subtilisin/kexin type 9; PPARs—peroxisome proliferator-activated receptors; TG—triglyceride; VLDL—very-low-density lipoprotein.
